# Network-based discovery of gene-miRNA interactions associated with hepatocellular carcinoma

**DOI:** 10.1007/s11845-026-04303-4

**Published:** 2026-04-02

**Authors:** Murat Isiyel, Hamid Ceylan

**Affiliations:** 1 Department of Molecular Biology and Genetics, Faculty of Science, Atatürk University, Erzurum, Türkiye 25240 Turkey; 2 East Anatolian High Technology Research and Application Center (DAYTAM), Atatürk University, Erzurum, Türkiye 25240 Turkey

**Keywords:** Liver, Hepatocellular carcinoma, DEGs, MiRNA, Bioinformatics

## Abstract

**Background:**

Hepatocellular carcinomaAQ1 (HCC), the most prevalent form of liver cancer, poses a significant health burden due to its aggressive nature and poor prognosis. This study employed a comprehensive integrative bioinformatics approach to uncover putative regulatory axes involved in HCC pathogenesis.

**Methods:**

Transcriptomic data from twelve GEO microarray datasets were analyzed to identify differentially expressed genes(DEGs) and differentially expressed miRNAs (DEmİRs) between tumor and adjacent non-tumorous liver tissues. To interpret the determined biological information and determine the hub genes, a protein-protein interaction (PPI)network was created, and gene ontology and pathway enrichment analysis were performed. Furthermore, in silico analyses were performed to verify the gene and protein expression levels of the identified hub genes and to assess their prognostic impact on HCC.

**Results:**

The findings identified three hub genes (AURKA, CCNB1, and KIF11) that were consistently shared across all datasets and were significantly associated with cancer progression and reduced overall survival. Pathway analysis indicated that the hub genes predominantly display protein kinase binding activity and participate in several pathways linked to cancer progression, including the p53 signaling pathway. Concurrently, three commonly downregulated miRNAs(hsa-miR-335-3p, hsa-miR-2355-5p, and hsa-miR-3163) were identified, all of which target the hub genes and contribute to their regulation.

**Conclusions:**

Collectively, these findings highlight bioinformatically prioritized gene–miRNA candidates associated with HCC and provide a rational basis for future experimental validation and functional studies.

**Supplementary Information:**

The online version contains supplementary material available at 10.1007/s11845-026-04303-4.

## Introduction

Liver cancer is the sixth most prevalent form of cancer globally, and hepatocellular carcinoma (HCC) is the most frequent subtype of liver cancer [1]. HCC is a frequently occurring malignant tumor of the digestive tract. HCC cells proliferate rapidly and exhibit significant vascular invasion and metastasis within and beyond the liver [2]. Research indicates that HCC development is heightened by metabolic factors, such as diabetes and obesity, along with demographic aspects, such as age, sex, and ethnicity [3]. Furthermore, the likelihood of HCC increases due to exposure to harmful substances, such as tobacco and alcohol, and persistent infections, such as hepatitis B (HBV) or hepatitis C virus (HCV) [4, 5]. Early identification of HCC is crucial because of its recurrence and high metastatic potential [6, 7]. Consequently, detecting molecular indicators for early diagnosis and treatment is crucial for lowering healthcare expenses and enhancing survival rates through appropriate treatment approaches [8].

MicroRNAs (miRNAs) are single-stranded small non-coding RNA fragments comprising approximately 19–25 nucleotides [9] that control gene expression at the post-transcriptional level. They are involved in significant biological processes, including programmed cell death, metabolism, cell growth, and differentiation [10]. Studies have proposed that miRNAs may act as tumor suppressors in various cancers, including prostate [11], colorectal [12], gastric [13], and breast cancers [14]. Evidence suggests that abnormal miRNA expression can influence HCC cell growth by directly interacting with cell cycle regulatory mechanisms [15]. Furthermore, miRNAs have been identified to perform a crucial function in HCC progression [16–18].

Advances in genome-level technologies, such as next-generation sequencing and microarray, have generated substantial RNA data, which are stored in open-access databases, such as Gene Expression Omnibus (GEO) and The Cancer Genome Atlas (TCGA) [19–21]. Using these datasets, differentially expressed genes (DEGs) related to various diseases, including HCC, and their functions were identified [22, 23]. However, variations in microarray platforms and sample sources may lead to inconsistent results [24]. Therefore, integrated bioinformatics analyses are essential for obtaining more dependable and precise results [25].

Recent high-throughput transcriptomic technologies have enabled systematic exploration of gene and microRNA expression alterations in HCC. However, findings derived from single-cohort studies are often limited by sample size, platform-specific effects, and population heterogeneity. To address these limitations, the present study integrates multiple independent GEO datasets, thereby reducing cohort-specific and platform-dependent bias and improving the robustness of identified molecular candidates. This study identified HCC-related hub genes and miRNAs by analyzing publicly available GEO microarray datasets using integrated bioinformatic approaches. The correlation between the identified DEGs and DEmiRs (differentially expressed microRNAs) was investigated to identify the potential biomarkers.

## Materials and methods

### Transcriptomics data

**Fig. 1:**
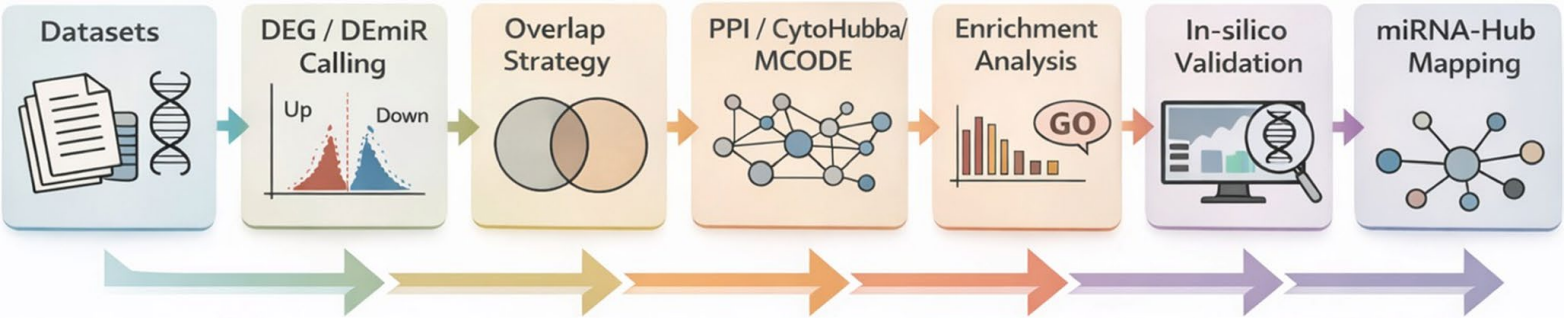
Overview of the integrative bioinformatics workflow used in this study

All analyses in this study, including DEG/DEmiR identification and all subsequent analyses (PPI network construction and enrichment analyses), were performed using publicly accessible bioinformatics tools with clearly defined and reproducible parameters to ensure methodological transparency. An overview of the complete analytical workflow employed in this study is presented in Fig. 1. Publicly accessible independent microarray datasets related to hepatocellular carcinoma were acquired from Gene Expression Omnibus (GEO: https://www.ncbi.nlm.nih.gov/geo/, accessed on May 9, 2025) [26]. A total of eleven mRNA expression datasets and one miRNA expression dataset, including 10 for DEGs and one for DEmiRs (filters were applied based on the study’s objectives, such as *Homo sapiens*, expression profiles, and HCC). Comprehensive features of the expression profiles are presented in Table 1.

**Table 1:** Features of the datasets used in this study

GEO Accession	Sample size	Platform	Profile	References
GSE216613	3 HCC tumor samples, 3 adjacent non-tumorous tissues	GPL24676	mRNA	[27]
GSE185799	3 HCC tumor samples, 3 adjacent non-tumorous tissues	GPL24676	mRNA	[28]
GSE159220	3 HCC tumor samples, 3 adjacent non-tumorous tissues	GPL11154	mRNA	[29]
GSE263134	5 HCC tumor samples, 5 adjacent non-tumorous tissues	GPL20301	mRNA	[30]
GSE242797	5 HCC tumor samples, 5 adjacent non-tumorous tissues	GPL28148	mRNA	[31]
GSE135631	15 HCC tumor samples, 15 adjacent non-tumorous tissues	GPL16791	mRNA	[32]
GSE169289	17 HCC tumor samples, 17 adjacent non-tumorous tissues	GPL16791	mRNA	[33]
GSE202069	41 HCC tumor samples, 25 adjacent non-tumorous tissues	GPL24676	mRNA	[34]
GSE214846	65 HCC tumor samples, 65 adjacent non-tumorous tissues	GPL24676	mRNA	[35]
GSE113617	68 HCC tumor samples, 10 adjacent non-tumorous tissues	GPL11154	mRNA	[36]
GSE64632	3 HCC tumor samples, 3 adjacent non-tumorous tissues	GPL18116	miRNA	[37]

*GEO* gene expression omnibus, *GPL* GEO Platform, *HCC* hepatocellular carcinoma

### DEGs and DEmiRs analysis

Samples were grouped as hepatocellular carcinoma tumor tissues and corresponding adjacent non-tumorous liver tissues according to the original dataset annotations. Differential expression analysis was performed using GEO2R (https://www.ncbi.nlm.nih.gov/geo2r, accessed on May 9, 2025). GEO2R default analysis settings were applied, including automatic log2 transformation of expression values when appropriate. Platform-specific annotation files provided by GEO were used to map probe identifiers to gene symbols. Statistical significance for DEG and DEmiR identification was determined using Benjamini–Hochberg false discovery rate (FDR)–adjusted p-values provided by GEO2R. Genes and miRNAs with an adjusted p-value < 0.05 were considered differentially expressed. Upregulated DEGs were defined as log2FC ≥ + 1 and downregulated DEGs as log2FC ≤ −1 (*p* < 0.05). Shared genes across all datasets were analyzed using a Venn diagram produced using the Multiple List Comparator web tool (http://molbiotools.com/listcompare.html, accessed on May 9, 2025). Predicted target genes of the differentially expressed miRNAs were obtained using TargetScanHuman (https://targetscan.org/vert_80/, accessed on May 9, 2025) [38]. Conserved targets were selected based on the cumulative weighted context + + score, and to enhance prediction confidence, only targets with a context + + score < − 0.2 were retained. After this filtering step, the predicted targets were intersected with the identified hub genes to construct putative miRNA–mRNA regulatory networks. Volcano plots were created using the Prism software (GraphPad Software, San Diego, CA, USA).

### Protein-protein interaction (PPI) network analysis

Protein–protein interaction (PPI) network analysis has been widely used to contextualize molecular targets and prioritize hub genes within broader functional and pathway frameworks [39–43]. In this study, a PPI network was constructed to evaluate interactions among genes with significantly altered expression levels in tumor tissues compared to adjacent non-tumorous liver tissues, thereby supporting downstream hub gene discovery and functional enrichment analyses [44, 45]. The PPI network was created using the Search Tool for Retrieval of Interacting Genes database (STRING: https://string-db.org/, accessed on May 9, 2025) [46]. A confidence score of ≥ 0.7 was applied to minimize false-positive interactions. This network was displayed and further analyzed using Cytoscape software version 3.10.3 [47]. Hub genes were ranked using the cytoHubba plugin by applying multiple topological algorithms, including Degree, Maximal Clique Centrality (MCC), Maximum Neighborhood Component (MNC), Edge Percolated Component (EPC), and Closeness. Genes consistently ranked among the top candidates across all applied algorithms were selected for further analysis. Highly interconnected network modules were identified using the MCODE plugin with the following parameters: degree cutoff = 2, node score cutoff = 0.2, k-core = 2, and maximum depth = 100. Hub genes were ultimately defined as the intersection between the top-ranked cytoHubba genes and genes located within significant MCODE clusters.

### Gene ontology and pathway enrichment analysis

Gene ontological enrichment analysis was conducted using the ToppFun (transcriptome, ontology, phenotype, proteome, and pharmacome annotations based gene list functional enrichment analysis) module of the ToppGene online bioinformatics resource (https://toppgene.cchmc.org/enrichment.jsp, accessed on May 9, 2025) [48]. Enrichment analysis of overlapping DEGs was conducted using Gene Ontology (GO) and Kyoto Encyclopedia of Genes and Genomes (KEGG) pathway analyses, which incorporated biological process (BP), molecular function (MF), and cellular component (CC) categories [49, 50].

### *In silico* comparison and validations

The Gene Expression Profiling Interactive Analysis (GEPIA; http://gepia.cancer-pku.cn/, accessed on May 9, 2025) platform [51] and the University of Alabama CANcer (UALCAN; https://ualcan.path.uab.edu/, accessed on May 9, 2025) interactive web portal [52] were used to compare the mRNA expression variations of hub genes between HCC tumor liver tissues and the corresponding adjacent non-tumorous liver tissues (histologically normal, tumor-adjacent, but without noticeable abnormalities) [53, 54] and to evaluate their prognostic significance. The interaction between the mRNA expression of hub genes and the clinical stage of liver cancer patients, as well as the protein levels encoded by hub genes, was also examined using UALCAN. Finally, the prognostic significance of the identified hub genes was evaluated using a publicly available survival analysis platform, Kaplan-Meier plotter(https://kmplot.com/analysis/, accessed on May 9, 2025) [55]. Overall survival was analyzed using univariate Kaplan–Meier methods. Patients were stratified into high- and low-expression groups according to the median expression level of each hub gene. No additional clinical covariates were included in the survival analysis.

## Results

### Determination of DEGs

**Fig. 2:**
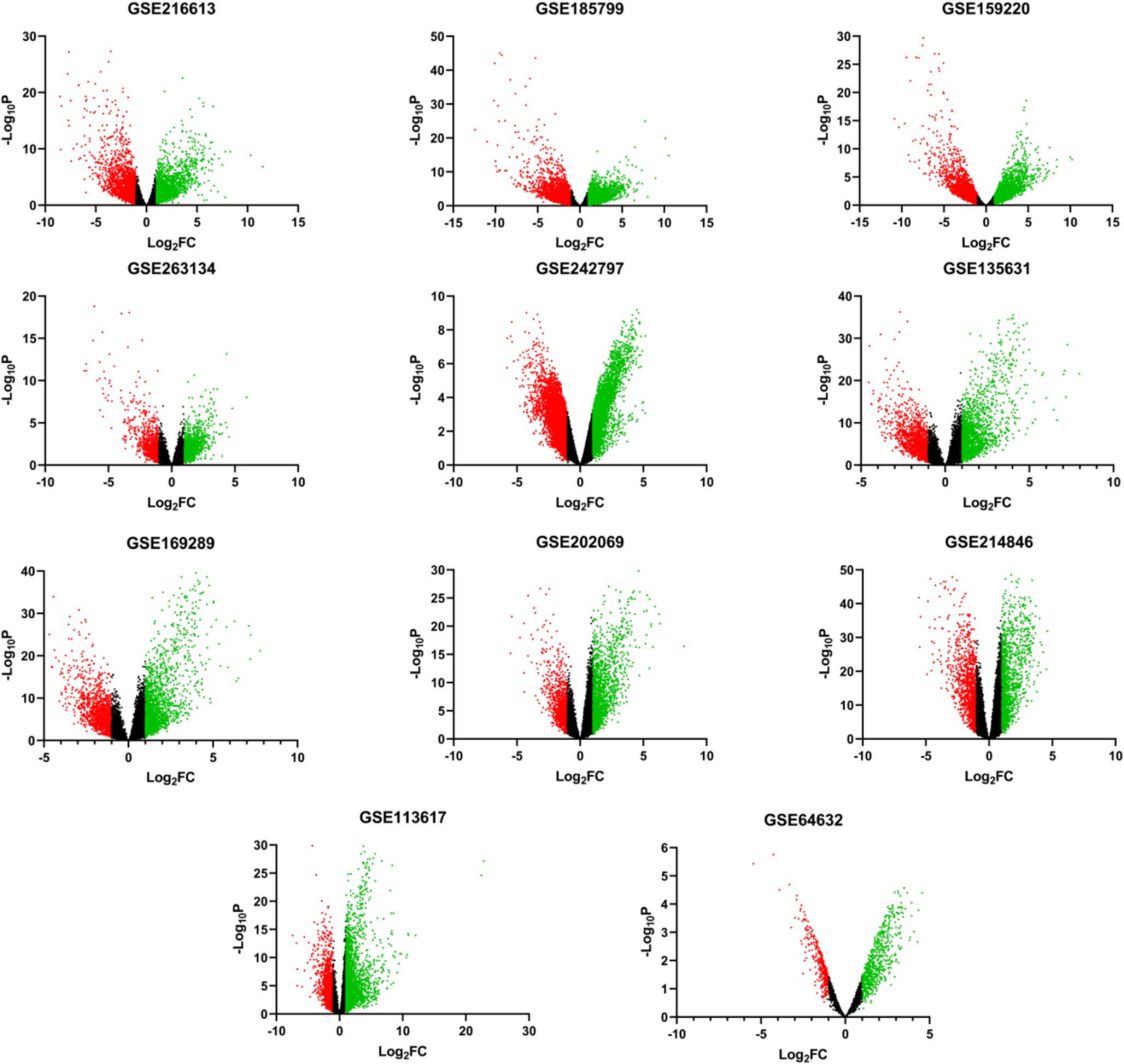
Volcano plots for the analyzed datasets revealed notable differences in expression between HCC and control samples. Upregulated DEGs were defined as log2FC ≥ + 1 and downregulated DEGs as log2FC ≤ −1 (*p* < 0.05). Green dots represent upregulated genes, red dots indicate downregulated genes, and black dots correspond to genes that fall outside the defined significance thresholds. FC: fold change; HCC: hepatocellular carcinoma

A total of 173 DEGs were identified, comprising 126 upregulated and 47 downregulated genes shared across all datasets. The distribution of DEGs and overlapping genes identified as a result of the analysis of the datasets is summarized in Tables S1 and S2. Volcano plots showing the distribution of DEGs created for each dataset are presented in Fig. 2.

### PPI network and cluster analysis

**Fig. 3:**
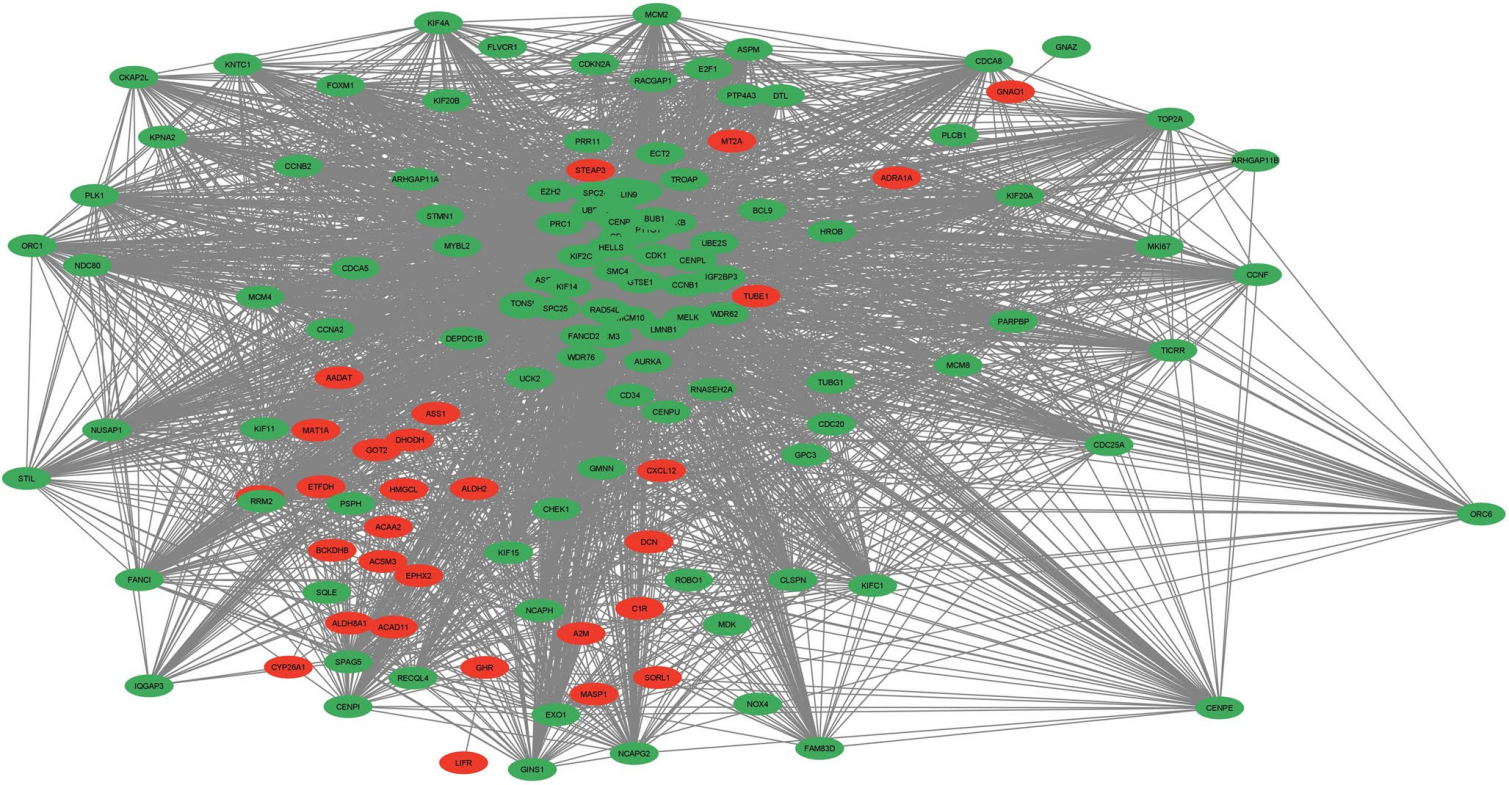
The protein–protein interaction (PPI) network constructed from the differentially expressed genes (DEGs). Green nodes represent upregulated DEGs, while red nodes indicate downregulated DEGs. Edges between nodes denote predicted or experimentally validated interactions, illustrating the complex molecular interplay and functional connectivity among DEGs involved in HCC pathogenesis.

**Fig. 4:**
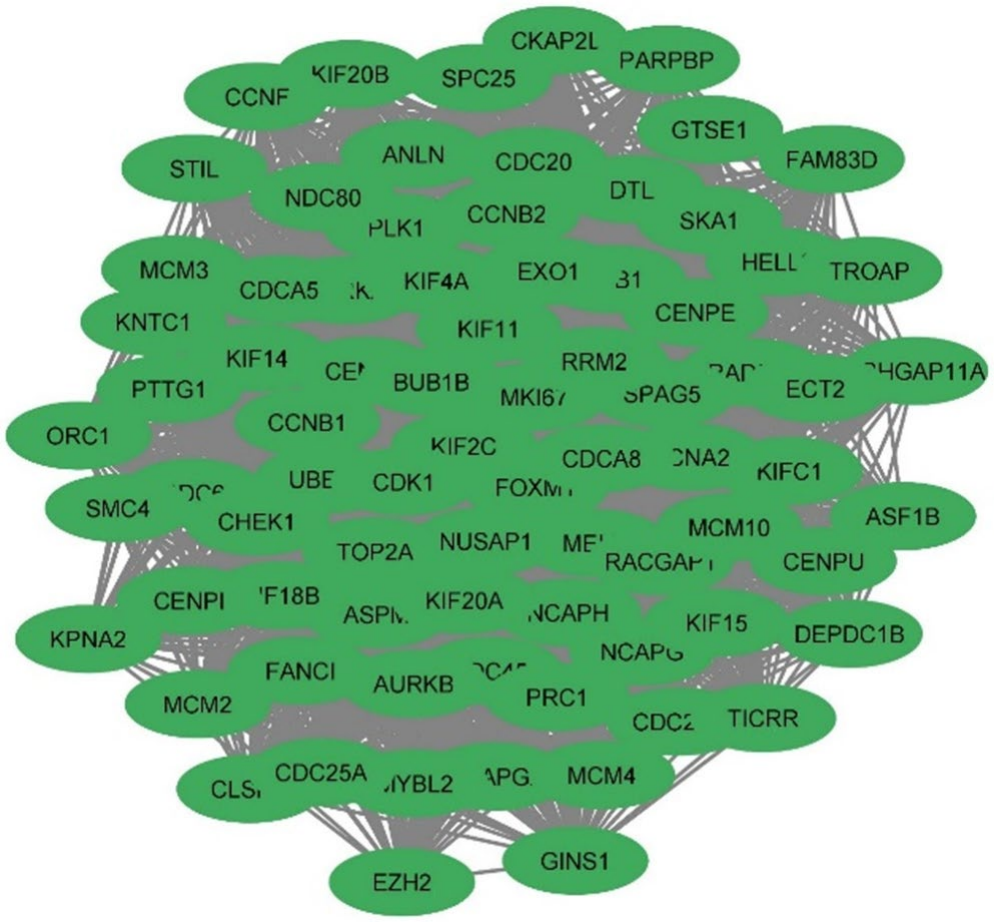
Module analysis of PPI network. Green nodes represent upregulated DEGs

**Fig. 5:**
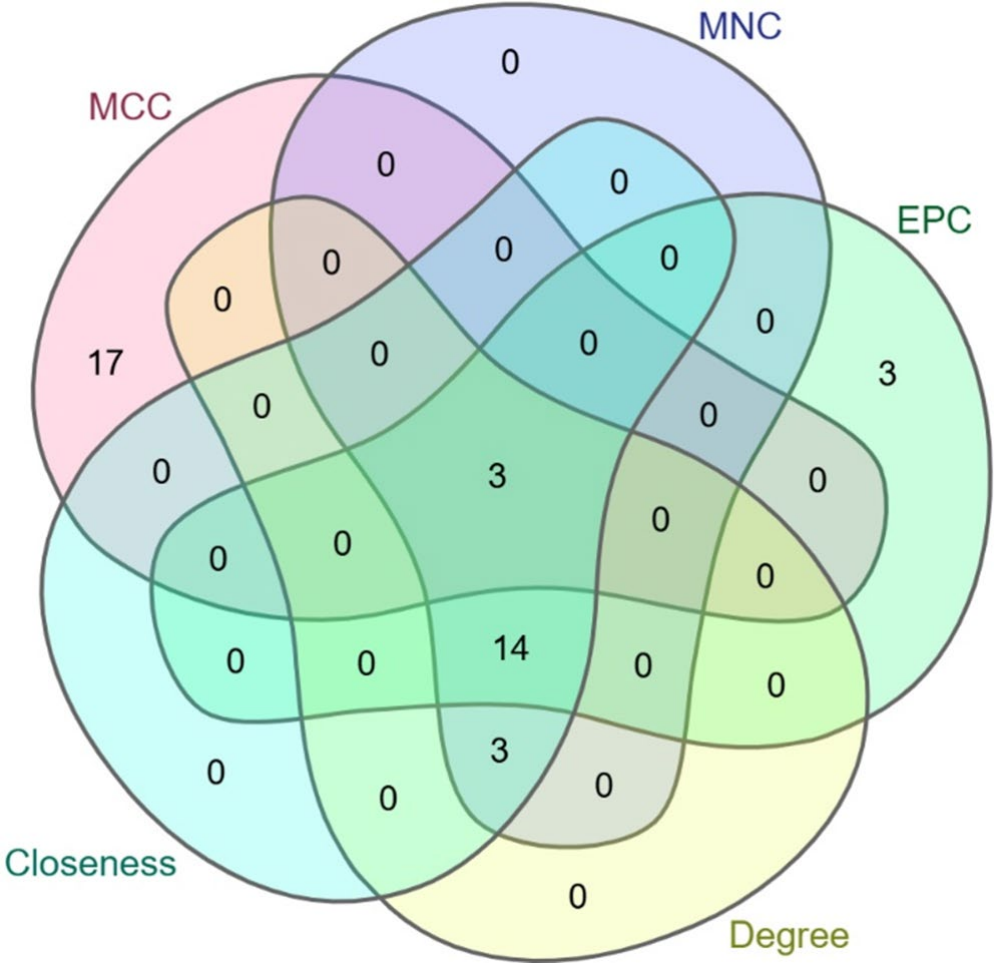
Venn diagram analysis demonstrating the overlapping DEGs among the 5 algorithms of CytoHubba. Maximal Clique Centrality; MCC, Maximum Neighborhood Component; MNC, Edge Percolated Component; (EPC)

A PPI network was constructed to analyze the interactions among genes with significantly altered expression in tumor tissues from individuals diagnosed with HCC compared with adjacent non-tumorous liver tissue samples. In the PPI network, which included 145 nodes and 3366 interactions (Fig. 3), a single significant cluster was identified based on its significance using the MCODE (clusters a given network based on topology to find densely connected regions) plugin (Fig. 4). The top 20 genes highlighted by the five topological algorithms in cytoHubba were selected (Table S3). Subsequently, hub gene candidates were identified by identifying the shared genes across all algorithms using a Venn diagram (Fig. 5). Ultimately, three upregulated genes (*AURKA*: Aurora kinases A, *CCNB1*: Cyclin B1, *and KIF1*: Kinesin family member 11) were identified as hub genes.

### Identification of HCC-associated DEmiRs and target gene selection

Upregulated and downregulated DEmiRs were examined using the GEO2R tool in the GSE64632 dataset. This analysis identified 396 upregulated and 231 downregulated miRNAs. Because all hub genes were upregulated, only downregulated miRNAs with predicted binding sites were considered. The genes targeted by all 231 downregulated miRNAs were determined using TargetScanHuman 8.0. Three of these miRNAs (*hsa-miR-335-3p*,* hsa-miR-2355-5p*, and *hsa-miR-3163*) were found to include all previously identified hub genes (*AURKA*, *CCNB1*, and *KIF11*) among their target genes.

### Gene ontology and pathway enrichment analysis

To explore the roles associated with 126 upregulated and 47 downregulated DEGs in liver hepatocellular carcinoma (LIHC), gene ontology (GO) and pathway (KEGG) enrichment analyses were conducted. The DEGs were classified into three functional groups: biological processes, cellular components, and molecular functions. Pathway analysis indicated that the hub genes predominantly displayed protein kinase binding activity and participated in various pathways closely linked to cancer progression, such as the p53 signaling pathway (Table S4).

### In silico validations

**Fig. 6:**
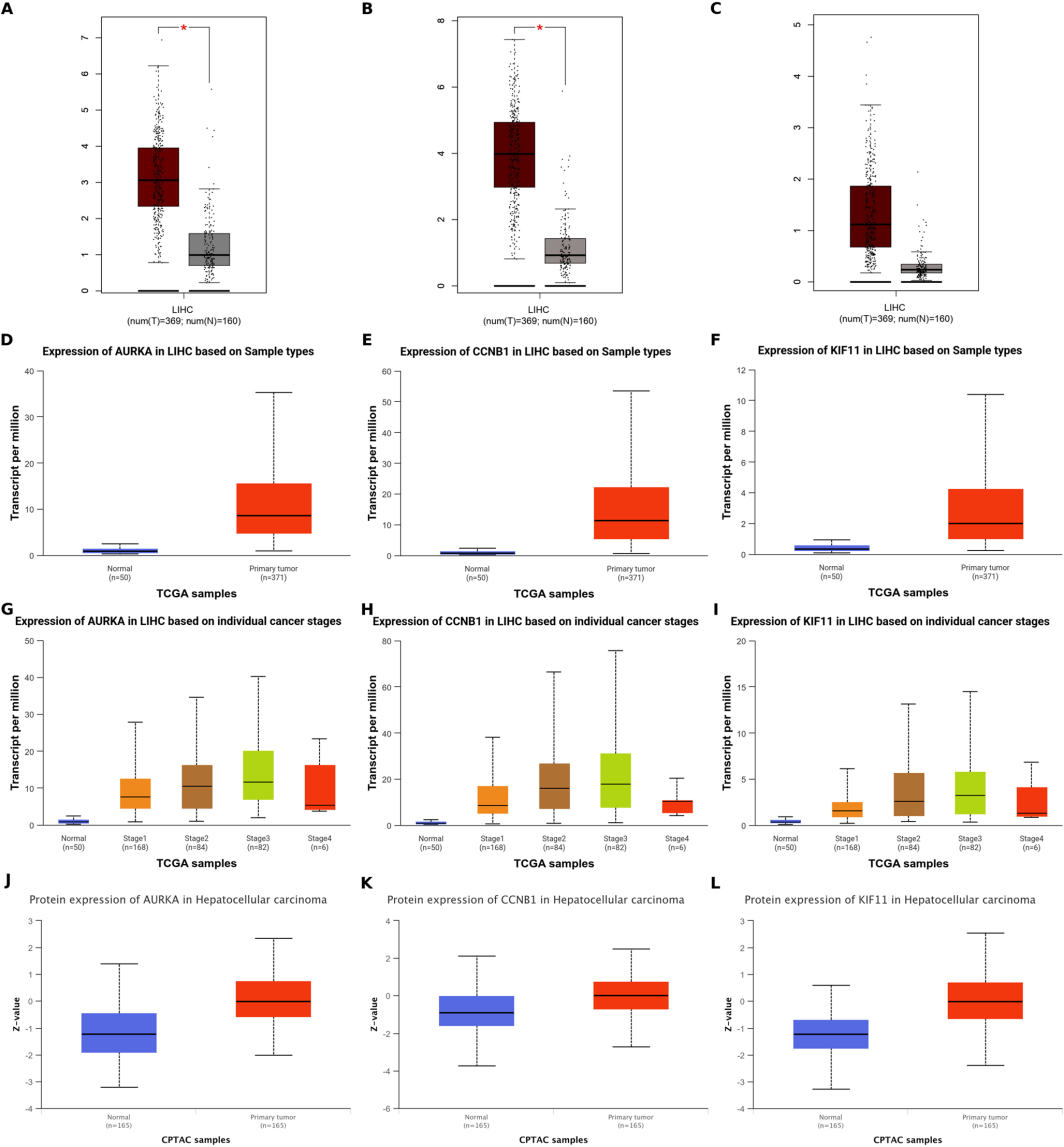
The mRNA and protein expression profiles of hub genes in normal liver tissues and LIHC tissues. The mRNA expression profiles of *AURKA* (**A**), *CCNB1* (**B**), and *KIF11* (**C**) in liver cancer within the GEPIA database. The mRNA expression profiles of *AURKA* (**D**), *CCNB1* (**E**), and *KIF11* (**F**) in liver cancer within the UALCAN database. The mRNA expression profiles of *AURKA* (**G**), *CCNB1* (**H**), and *KIF11* (**I**) in liver cancer patients in different cancer stages. Protein expression profiles of AURKA (**J**), CCNB1 (**K**), and KIF11 (**L**) in liver cancer within the UALCAN database. *AURKA*: Aurora kinases A, *CCNB1*: Cyclin B1, and *KIF11*: Kinesin family member 11. LIHC: liver hepatocellular carcinoma, TCGA: The Cancer Genome Atlas

**Fig. 7:**
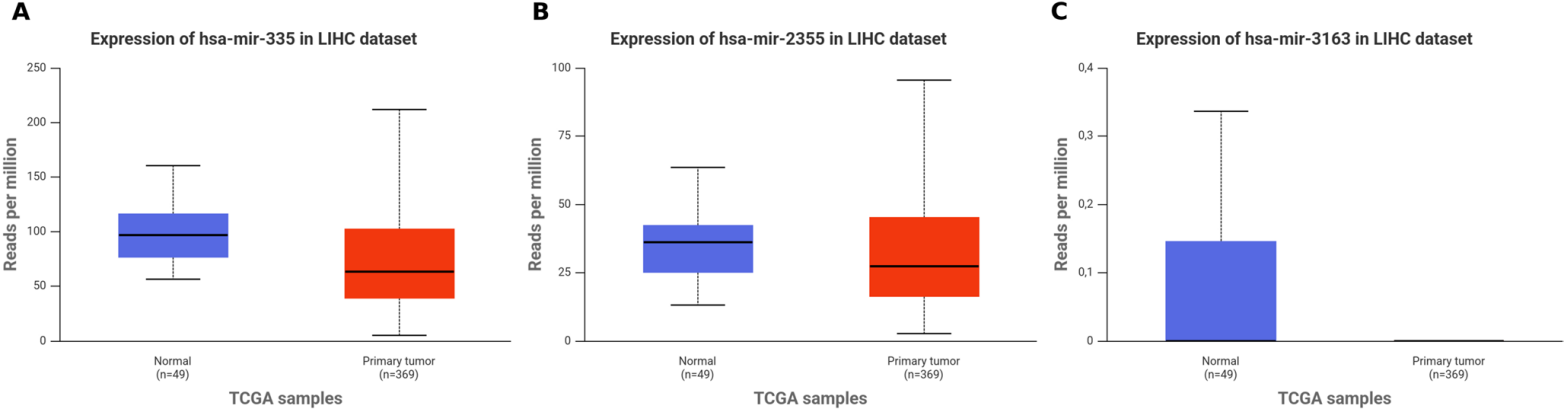
DEmiRs expression profiles of *hsa-miR-335-3p* (**A**), *hsa-miR-2355-5p* (**B**), and *hsa-miR-3163* (**C**) in normal liver tissues and LIHC tissues within the UALCAN database. LIHC: liver hepatocellular carcinoma, TCGA: The Cancer Genome Atlas

The expression differences of the identified hub genes between liver tissues from patients diagnosed with HCC and adjacent non-tumorous liver tissues were validated using the GEPIA and UALCAN databases. Consistent with the findings from the GEO dataset analysis, the mRNA expression profiles of *AURKA*, *CCNB1*, and *KIF11* were upregulated in HCC tumor tissues compared to adjacent non-tumorous liver tissues (Fig. 6A-F). Additionally, the expression variations of hub genes at different stages of LIHC were examined using the UALCAN database. The data obtained demonstrated that the mRNA expression of *AURKA*, *CCNB1*, and *KIF11* continued to rise in the advanced stages of LIHC (Fig. 6G, H, and I). The protein expression levels of AURKA, CCNB1, and KIF11 aligned with the mRNA expression levels between the liver tissues of HCC patients and adjacent non-tumorous liver tissues (Fig. 6J, K, and L). The expression levels of the identified DEmiRs were evaluated using the UALCAN platform. The results indicated that all miRNAs were downregulated (Fig. 7A, B, and C), and these findings were associated with the upregulation of hub genes.

**Fig. 8:**
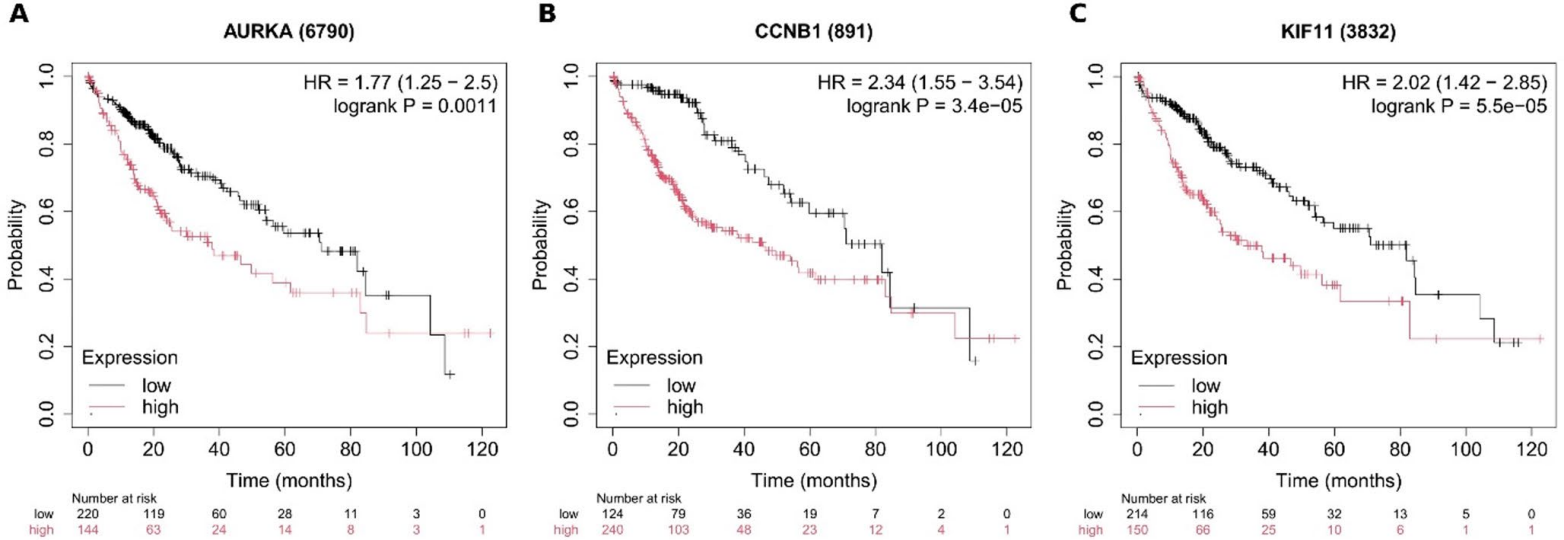
Overall survival (OS) analyses of the hub genes in patients with HCC. (**A**) *AURKA*, (**B**) *CCNB1*, and (**C**) *KIF11*

To analyze the prognostic impact of hub genes, an overall survival (OS) analysis of LIHC patients was conducted using the Kaplan-Meier method. The findings suggested that increased expression of *AURKA*, *CCNB1*, and *KIF11* was linked to poorer OS rates in patients with LIHC (Fig. 8). Detailed information on the prognostic value of the identified hub genes for HCC is provided in Table 2. In conclusion, these results imply that the identified hub genes could function as bioinformatically prioritized candidates for prognostication in liver hepatocellular carcinoma patients.

**Table 2:** Detailed information on the prognostic value ​​of the identified hub genes in HCC

Gene	Modulation in HCC	Probe-ID	HR	CI	Log-rank *p*-value	Median survival low (mo)	Median survival high (mo)
*AURKA*	Up	6790	1.77	1.25–2.5	0.0011	71	37.8
*KIF11*	Up	3832	2.02	1.42–2.85	5.5e-05	81.9	33.5
*CCNB1*	Up	891	2.34	1.55–3.54	3.4e-05	81.9	45.7

## Discussion

HCC is the leading cause of liver cancer incidence and mortality, accounting for the majority of the deaths. Despite advanced early detection and preventive screening approaches, the complex processes that contribute to HCC development and progression make it difficult to determine effective treatment approaches. Therefore, it is vital to identify biomarkers that can help in effective diagnosis and treatment strategies and to investigate their roles in the fight against the disease. In recent years, with the development of tools that allow more efficient integrated bioinformatics analysis, it has become possible to identify prominent bioinformatically prioritized candidates associated with complex diseases, such as cancer. In this study, DEGs were identified by intersecting eleven independent GEO mRNA expression datasets using a Venn diagram–based approach. Although this strategy is inherently conservative, it was intentionally adopted to prioritize robust and reproducible expression changes shared across multiple cohorts and microarray platforms. By focusing on consistently altered genes, this approach minimizes platform-specific noise and cohort-dependent bias commonly observed in single-dataset analyses. However, we acknowledge that such an intersection-based strategy may lead to the exclusion of biologically relevant genes that are specific to certain cohorts or platforms. Therefore, while enhancing robustness, this approach involves a trade-off between stringency and sensitivity, which should be considered when interpreting the results. Findings of the present study suggest that *AURKA*, *CCNB1*, and *KIF11* are candidate hub genes and that *hsa-miR-335-3p*,* hsa-miR-2355-5p*, and *hsa-miR-3163* are critical miRNAs involved in the regulation of these genes.

Aurora kinases A, B, and C (AURKA, AURKB, and AURKC) are crucial regulators of mitotic cell division. Despite similarities in kinase activity and protein structure, AURKA is distinct because of its vital cellular roles, including epithelial-mesenchymal transition (EMT) [56], DNA damage repair [57], and cell migration and invasion [58, 59]. AURKA overexpression has been linked to the initiation and progression of cancer. It affects key cellular pathways that regulate tumor invasion, migration, survival, and proliferation [60, 61]. AURKA expression is elevated in different cancer types, including head and neck [62], colorectal [63], bladder [64], breast [65], and HCC [66, 67]. Numerous studies have reported that HCC patients with high *AURKA* expression have shorter overall survival (OS) rates, whereas patients with low *AURKA* expression tend to have longer OS [68–71]. Jeng et al. highlighted that AURKA overexpression plays a significant role in the development and progression of HCC [72]. Chen et al. suggested that AURKA might be closely linked to tumor recurrence. Based on these findings, AURKA is anticipated to be a potential therapeutic target [73].

Over time, B-type cyclins have gained significant attention because of their upregulation during the G2/M phase, which facilitates mitosis [74]. Cyclin B1 (CCNB1) is one of the key B-type cyclins involved in promoting mitosis. Its expression rises and falls at different stages of the cell cycle [75]. Recent studies have indicated that CCNB1 is highly expressed in multiple cancer types, such as lung cancer [76], breast cancer [77], melanoma [78], and HCC [75]. In a study by Gu et al. targeting CCNB1 in the HCC-associated SMMC-7721 cell line, they found that knocking out CCNB1 resulted in the suppression of the invasiveness and migration of cancer cells [79]. Zhuang et al. reported that high CCNB1 expression is associated with poorer overall survival (OS) in HCC patients. They also suggested that CCNB1 may serve as a reliable biomarker and a potential therapeutic target [80]. Therefore, modulation of *CCNB1* expression represents a promising therapeutic avenue to reduce tumor invasiveness and improve survival outcomes, especially in hepatocellular carcinoma.

Kinesin family member 11 (KIF11), also referred to as EG5, is a motor protein comprising 1056 amino acids [81, 82]. KIF11 plays a crucial role in the transport of secreted proteins [83] and in mitosis [84]. Genetic abnormalities resulting from KIF11 defects contribute to cancer progression by enhancing invasion and metastasis [85]. KIF11 is broadly expressed in bone marrow, lymph nodes, and other healthy tissues [86]. Abnormal KIF11 expression disrupts normal cell division and enhances tumor cell growth and migration in various cancer types [87–89]. Recent studies have demonstrated that *KIF11* is overexpressed in gastric cancer [90], non-small cell lung cancer [91], prostate cancer [92], oral cancer [93], and HCC [86]. High levels of KIF11 expression in HCC are strongly linked to increased malignancy, suggesting that KIF11 could be a potential therapeutic target [94]. Modulating the abnormal overexpression of KIF11 is especially important in hepatocellular carcinoma, where its levels correlate with increased malignancy. This relationship underscores the critical role of KIF11 in tumor progression and its potential as a therapeutic target.

miRNAs are small RNA fragments that modulate gene expression at the post-transcriptional stage, play important roles in the development and establishment of cellular identity [95], and are estimated to modulate the translation of approximately 30% of the protein-coding genes [96]. Functional defects in miRNA pathways closely affect cancer-associated cellular roles, including apoptosis, differentiation, metastasis, and telomere maintenance [97–99]. It has been reported that some miRNAs display varying expression levels in cancer and may affect carcinogenesis, metastasis, and cellular transformation by acting as tumor suppressors or oncogenes [95]. Considering the three target miRNAs identified, similar results have been observed in studies conducted on different cancer types. Qiu et al. (2021) suggested that *hsa-miR-3163* is related to malignant tumor inhibition in breast cancer and that *hsa-miR-3163* and CCNB1 may serve as candidate biomarkers [100]. Yang et al. stated that overexpression of *hsa-miR-3163* could increase the sensitivity of hepatocellular carcinoma cells to molecular targeted agents [101]. *miR-335-5p* has been indicated to function either as a tumor suppressor or as an oncomiR in various types of cancers [102]. Downregulation of *miR-335-5p* has been reported to be associated with abnormal DNA methylation in different cancer types, particularly gastric cancer [103] and HCC [104, 105]. Modulating miRNA expression is a promising strategy for regulating carcinogenesis by influencing key cellular processes such as apoptosis, differentiation, metastasis, and telomere maintenance. This approach may also provide valuable opportunities for both diagnosis and therapy across different cancer types.

Several limitations of this study should be acknowledged. First, the analyses relied on publicly available microarray datasets, which may be influenced by platform-specific bias and technical heterogeneity, despite the use of multiple independent datasets. Second, the intersection-based DEG selection strategy is inherently conservative and may exclude cohort- or platform-specific but biologically relevant genes. Third, miRNA–target relationships were inferred using in silico prediction tools and may not fully reflect experimentally validated interactions. Finally, the absence of experimental validation, such as qPCR, Western blotting, or functional assays, represents a limitation of the present work. Further experimental studies are therefore required to validate these bioinformatic findings.

## Conclusion

In this study, hub genes associated with HCC and linked to poor prognosis, as well as common miRNAs potentially regulating their expression, were identified through bioinformatics analyses. The findings contribute to a better understanding of the molecular mechanisms underlying HCC and provide a valuable basis for the identification of potential diagnostic and prognostic biomarkers. However, further functional studies and comprehensive validation analyses in human subjects are required to clarify the clinical relevance and biological roles of these candidate genes and miRNAs. As with all in silico analyses, experimental validation in patient-derived samples is required to confirm these findings.

## Supplementary Information

Below is the link to the electronic supplementary material.

Supplementary Material 1 (DOCX 36.1 KB)

## Data Availability

All relevant data analyzed during the current study are available in the GEO repository.

## References

[1] Rumgay H, Ferlay J, de Martel C, Georges D, Ibrahim AS, Zheng R et al (2022) Global, regional and national burden of primary liver cancer by subtype. Eur J Cancer 161:108–118 34942552 10.1016/j.ejca.2021.11.023

[2] Siegel RL, Miller KD, Wagle NS, Jemal A (2023) Cancer statistics, 2023. CA Cancer J Clin 73(1):17–48. 10.3322/caac.21763 36633525 10.3322/caac.21763

[3] Vogel A, Meyer T, Sapisochin G, Salem R, Saborowski A (2022) Hepatocellular carcinoma. Lancet 400(10360):1345–1362. 10.1016/S0140-6736(22)01200-4 36084663 10.1016/S0140-6736(22)01200-4

[4] Galle PR, Forner A, Llovet JM, Mazzaferro V, Piscaglia F, Raoul J-L et al (2018) EASL clinical practice guidelines: management of hepatocellular carcinoma. J Hepatol 69(1):182–236 29628281 10.1016/j.jhep.2018.03.019

[5] Revoredo S, Del Fabbro E (2023) Hepatocellular carcinoma and sarcopenia: a narrative review. Ann Palliat Med 12(6):1295–309. 10.21037/apm-23-332 37872128 10.21037/apm-23-332

[6] Liu Y, Veeraraghavan V, Pinkerton M, Fu J, Douglas MW, George J et al (2021) Viral biomarkers for hepatitis B virus-related hepatocellular carcinoma occurrence and recurrence. Front Microbiol 12:665201. 10.3389/fmicb.2021.665201 34194408 10.3389/fmicb.2021.665201 PMC8236856

[7] Kalaki NS, Ahmadzadeh M, Mansouri A, Saberiyan M, Karbalaie Niya MH (2024) Identification of hub genes and pathways in hepatitis B virus-associated hepatocellular carcinoma: a comprehensive in silico study. Health Sci Rep 7(6):e2185. 10.1002/hsr2.2185 38895552 10.1002/hsr2.2185 PMC11183944

[8] Sun J, Zhang Z, Cai J, Li X, Xu X (2024) Identification of hub genes in liver hepatocellular carcinoma based on weighted gene co-expression network analysis. Biochem Genet. 10.1007/s10528-024-10803-8 38683466 10.1007/s10528-024-10803-8 PMC12144059

[9] Lou W, Liu J, Gao Y, Zhong G, Ding B, Xu L et al (2018) MicroRNA regulation of liver cancer stem cells. Am J Cancer Res 8(7):1126–1141 30094089 PMC6079154

[10] Sticht C, De La Torre C, Parveen A, Gretz N (2018) miRWalk: an online resource for prediction of microRNA binding sites. PLoS One 13(10):e0206239. 10.1371/journal.pone.0206239 30335862 10.1371/journal.pone.0206239 PMC6193719

[11] Jackson BL, Grabowska A, Ratan HL (2014) MicroRNA in prostate cancer: functional importance and potential as circulating biomarkers. BMC Cancer 14:930. 10.1186/1471-2407-14-930 25496077 10.1186/1471-2407-14-930 PMC4295407

[12] Liu N, Jiang F, Han XY, Li M, Chen WJ, Liu QC et al (2018) MiRNA-155 promotes the invasion of colorectal cancer SW-480 cells through regulating the Wnt/beta-catenin. Eur Rev Med Pharmacol Sci 22(1):101 – 9. 10.26355/eurrev_201801_14106 29364476

[13] Schmitt AM, Garcia JT, Hung T, Flynn RA, Shen Y, Qu K et al (2016) An inducible long noncoding RNA amplifies DNA damage signaling. Nat Genet 48(11):1370–6. 10.1038/ng.3673 27668660 10.1038/ng.3673 PMC5083181

[14] Bai X, Han G, Liu Y, Jiang H, He Q (2018) MiRNA-20a-5p promotes the growth of triple-negative breast cancer cells through targeting RUNX3. Biomed Pharmacother 103:1482–9. 10.1016/j.biopha.2018.04.165 29864933 10.1016/j.biopha.2018.04.165

[15] Huang JL, Zheng L, Hu YW, Wang Q (2014) Characteristics of long non-coding RNA and its relation to hepatocellular carcinoma. Carcinogenesis 35(3):507–14. 10.1093/carcin/bgt405 24296588 10.1093/carcin/bgt405

[16] Li Q, Li S, Wu Y, Gao F (2017) MiRNA-708 functions as a tumour suppressor in hepatocellular carcinoma by targeting SMAD3. Oncol Lett 14(2):2552–2558. 10.3892/ol.2017.6429 28789462 10.3892/ol.2017.6429 PMC5530066

[17] Pan XP, Wang HX, Tong DM, Li Y, Huang LH, Wang C (2017) MiRNA-370 acts as a tumor suppressor via the downregulation of PIM1 in hepatocellular carcinoma. Eur Rev Med Pharmacol Sci 21(6):1254–1263 28387905

[18] Yu Q, Yang X, Duan W, Li C, Luo Y, Lu S (2017) MiRNA-346 promotes proliferation, migration and invasion in liver cancer. Oncol Lett 14(3):3255–3260. 10.3892/ol.2017.6561 28927074 10.3892/ol.2017.6561 PMC5587998

[19] Ceylan H (2021) Identification of hub genes associated with obesity-induced hepatocellular carcinoma risk based on integrated bioinformatics analysis. Med Oncol 38(6):63. 10.1007/s12032-021-01510-0 33900477 10.1007/s12032-021-01510-0

[20] Tosun H, Karadas H, Ceylan H (2024) “ Bioinformatics-based identification of hepatocellular carcinoma-associated hub genes and assessment of the restorative effect of tannic acid in rat liver exposed to monosodium glutamate.” Cancer Med-Us. 10.1002/cam4.7404

[21] Isiyel M, Ceylan H, Demir Y (2025) Integrated bioinformatics and molecular docking identify CCNB1, CDK1, and CYP1A2 as therapeutic targets of phytochemicals in hepatocellular carcinoma. Naunyn Schmiedebergs Arch Pharmacol. 10.1007/s00210-025-04729-0 41099846 10.1007/s00210-025-04729-0

[22] Clough E (2016) T <>Barrett The Gene Expression Omnibus Database. Methods Mol Biol 1418 93–110 10.1007/978-1-4939-3578-9_5 27008011 10.1007/978-1-4939-3578-9_5 PMC4944384

[23] Lee SC, Tan HT, Chung MC (2014) “ Prognostic biomarkers for prediction of recurrence of hepatocellular carcinoma: current status and future prospects.” World J Gastroenterol 20(12):3112–24. 10.3748/wjg.v20.i12.3112 24696598 10.3748/wjg.v20.i12.3112 PMC3964383

[24] Zhao X, Sun S, Zeng X, Cui L (2018) Expression profiles analysis identifies a novel three-mRNA signature to predict overall survival in oral squamous cell carcinoma. Am J Cancer Res 8(3):450–461 29637000 PMC5883095

[25] Ma X, Zhou L, Zheng S (2020) “ Transcriptome analysis revealed key prognostic genes and microRNAs in hepatocellular carcinoma.” PeerJ 8:e8930. 10.7717/peerj.8930 32296612 10.7717/peerj.8930 PMC7150540

[26] Edgar R, Domrachev M, Lash AE (2002) “ Gene expression omnibus: NCBI gene expression and hybridization array data repository.” Nucleic Acids Res 30(1):207–10. 10.1093/nar/30.1.207 11752295 10.1093/nar/30.1.207 PMC99122

[27] Komatsu J, Cico A, Poncin R, Le Bohec M, Morf J, Lipin S et al (2023) RevGel-seq: instrument-free single-cell RNA sequencing using a reversible hydrogel for cell-specific barcoding. Sci Rep 13(1):4866. 10.1038/s41598-023-31915-y 36964177 10.1038/s41598-023-31915-y PMC10039079

[28] Zhu P, Pei Y, Yu J, Ding W, Yang Y, Liu F et al (2023) High-throughput sequencing approach for the identification of lncRNA biomarkers in hepatocellular carcinoma and revealing the effect of ZFAS1/miR-150-5p on hepatocellular carcinoma progression. PeerJ 11:e14891. 10.7717/peerj.14891 36855431 10.7717/peerj.14891 PMC9968462

[29] Zhang Y, Li J, Cui Q, Hu P, Hu S, Qian Y (2022) Circular RNA hsa_circ_0006091 as a novel biomarker for hepatocellular carcinoma. Bioengineered 13(2):1988–2003. 10.1080/21655979.2021.2006952 35068348 10.1080/21655979.2021.2006952 PMC8973770

[30] Wei X, Si A, Zhao S, Fu Y, Li J, Aishanjiang K et al (2025) CircUCK2(2,3) promotes cancer progression and enhances synergistic cytotoxicity of lenvatinib with EGFR inhibitors via activating CNIH4–TGFα–EGFR signaling. Cell Mol Biol Lett 30(1):15. 10.1186/s11658-025-00690-1 39885395 10.1186/s11658-025-00690-1 PMC11781035

[31] Liu Y, Dong Z, Chen W, Chen L, Ju L, Cai W et al (2023) Construction of a ceRNA regulatory network to explore potential pathogenesis mechanisms involved in human hepatocellular carcinoma. Sci Rep 13(1):22058. 10.1038/s41598-023-47374-4 38086834 10.1038/s41598-023-47374-4 PMC10716167

[32] Yin J, Chen X, Li N, Han X, Liu W, Pu R et al (2021) Compartmentalized evolution of hepatitis B virus contributes differently to the prognosis of hepatocellular carcinoma. Carcinogenesis 42(3):461–70. 10.1093/carcin/bgaa127 33247709 10.1093/carcin/bgaa127

[33] Chen X, Wu T, Xian L, Ma L, Li N, Liu W et al (2023) CircGLS2 inhibits hepatocellular carcinoma recurrence via regulating hsa-miR-222-3p-PTEN-AKT signaling. Signal Transduct Target Ther 8(1):67. 10.1038/s41392-022-01275-6 36797228 10.1038/s41392-022-01275-6 PMC9935627

[34] Li B, Li Y, Zhou H, Xu Y, Cao Y, Cheng C et al (2024) Multiomics identifies metabolic subtypes based on fatty acid degradation allocating personalized treatment in hepatocellular carcinoma. Hepatology 79(2):289–306. 10.1097/HEP.0000000000000553 37540187 10.1097/HEP.0000000000000553 PMC10789383

[35] Long M, Zhou Z, Wei X, Lin Q, Qiu M, Zhou Y et al (2022) A novel risk score based on immune-related genes for hepatocellular carcinoma as a reliable prognostic biomarker and correlated with immune infiltration. Front Immunol 13:1023349. 10.3389/fimmu.2022.1023349 36353638 10.3389/fimmu.2022.1023349 PMC9637590

[36] Yoon S, Choi JH, Shah M, Kwon SM, Yang J, Park YN et al (2021) USO1 isoforms differentially promote liver cancer progression by dysregulating the ER-Golgi network. Carcinogenesis 42(9):1208–20. 10.1093/carcin/bgab067 34293111 10.1093/carcin/bgab067

[37] Peng H, Ishida M, Li L, Saito A, Kamiya A, Hamilton JP et al (2015) Pseudogene INTS6P1 regulates its cognate gene INTS6 through competitive binding of miR-17-5p in hepatocellular carcinoma. Oncotarget 6(8):5666 – 77. 10.18632/oncotarget.3290 PMC4467393 25686840

[38] McGeary SE, Lin KS, Shi CY, Pham TM, Bisaria N, Kelley GM et al (2019) “ The biochemical basis of microRNA targeting efficacy.” Science. 10.1126/science.aav1741 31806698 10.1126/science.aav1741 PMC7051167

[39] Gulec O, Duran HE, Arslan M, Yildiztekin G, Ece A, Turkes C (2025) “ Chalcone-inspired indole, carbazole, and phenothiazine hybrids as potent aldose reductase inhibitors with selective anticancer potential: rational design, synthesis, and multi-level characterization.” Bioorg Chem 164:108861. 10.1016/j.bioorg.2025.108861 40789257 10.1016/j.bioorg.2025.108861

[40] Gundogdu S, Duran HE, Arslan M, Cetinkaya BD, Turkes C (2025) “ Fluorenyl-phthalimide hybrids as potent aldose reductase inhibitors with selective anticancer activity: rational design, synthesis, and molecular insights.” Bioorg Chem 163:108689. 10.1016/j.bioorg.2025.108689 40570667 10.1016/j.bioorg.2025.108689

[41] Hoti D, Nixha AR, Duran HE, Arslan M, Yildiztekin G, Ece A et al (2025) “ Phthalimide-benzoic acid hybrids as potent aldose reductase inhibitors: synthesis, enzymatic kinetics, and in silico characterization.” Bioorg Med Chem 131:118416. 10.1016/j.bmc.2025.118416 41027295 10.1016/j.bmc.2025.118416

[42] Turkes C (2026) “ Machine learning-guided repurposing of FDA-approved quinolones as dual cholinesterase inhibitors: a multi-level docking, molecular dynamics, DFT, and SHAP-based analysis.” J Mol Graph Model 143:109259. 10.1016/j.jmgm.2025.109259 41412008 10.1016/j.jmgm.2025.109259

[43] Zognjani B, Nixha AR, Duran HE, Arslan M, Yildiztekin G, Ece A et al (2025) N-substituted phthalimide-carboxylic acid hybrids as dual-targeted aldose reductase inhibitors: Synthesis, mechanistic insights, and cancer-relevant profiling. Bioorg Chem 163:108788. 10.1016/j.bioorg.2025.108788 40716159

[44] Ceylan H (2021) A bioinformatics approach for identifying potential molecular mechanisms and key genes involved in COVID-19 associated cardiac remodeling. Gene Rep 24:101246. 10.1016/j.genrep.2021.101246 34131597 10.1016/j.genrep.2021.101246 PMC8192842

[45] Ceylan H (2022) “ Integrated bioinformatics analysis to identify alternative therapeutic targets for Alzheimer’s disease: insights from a synaptic machinery perspective.” J Mol Neurosci 72(2):273–86. 10.1007/s12031-021-01893-9 34414562 10.1007/s12031-021-01893-9

[46] Szklarczyk D, Franceschini A, Wyder S, Forslund K, Heller D, Huerta-Cepas J et al (2015) STRING v10: protein-protein interaction networks, integrated over the tree of life. Nucleic Acids Res 43(Database issue):D447–D452. 10.1093/nar/gku1003 25352553 10.1093/nar/gku1003 PMC4383874

[47] Shannon P, Markiel A, Ozier O, Baliga NS, Wang JT, Ramage D et al (2003) “ Cytoscape: a software environment for integrated models of biomolecular interaction networks.” Genome Res 13(11):2498–504. 10.1101/gr.1239303 14597658 10.1101/gr.1239303 PMC403769

[48] Chen J, Bardes EE, Aronow BJ, Jegga AG (2009) “ ToppGene suite for gene list enrichment analysis and candidate gene prioritization.” Nucleic Acids Res 37(Web Server):W305-11. 10.1093/nar/gkp427 19465376 10.1093/nar/gkp427 PMC2703978

[49] Karadas H, Tosun H, Ceylan H (2024) Identification of dilated cardiomyopathy-linked key genes by bioinformatics methods and evaluating the impact of tannic acid and monosodium glutamate in rats. Biotechnol Appl Biochem. 10.1002/bab.2670 39318238 10.1002/bab.2670 PMC11975261

[50] Isiyel M, Ceylan H, Demir Y (2026) Bioinformatics-based discovery of therapeutic targets in cadmium-induced lung adenocarcinoma: the role of oxyresveratrol. Biol Trace Elem Res 204(2):1068–1083. 10.1007/s12011-025-04730-x 40610695 10.1007/s12011-025-04730-x PMC12847123

[51] Tang Z, Kang B, Li C, Chen T, Zhang Z (2019) “ GEPIA2: an enhanced web server for large-scale expression profiling and interactive analysis.” Nucleic Acids Res 47(W1):W556–W60. 10.1093/nar/gkz430 31114875 10.1093/nar/gkz430 PMC6602440

[52] Chandrashekar DS, Bashel B, Balasubramanya SAH, Creighton CJ, Ponce-Rodriguez I, Chakravarthi B et al (2017) UALCAN: A Portal for Facilitating Tumor Subgroup Gene Expression and Survival Analyses. Neoplasia 19(8):649–658. 10.1016/j.neo.2017.05.002 28732212 10.1016/j.neo.2017.05.002 PMC5516091

[53] Caputo WL, de Souza MC, Basso CR, Pedrosa VA, Seiva FRF (2023) “ Comprehensive profiling and therapeutic insights into differentially expressed genes in hepatocellular carcinoma.” Cancers (Basel). 10.3390/cancers15235653 38067357 10.3390/cancers15235653 PMC10705715

[54] Aran D, Camarda R, Odegaard J, Paik H, Oskotsky B, Krings G et al (2017) “ Comprehensive analysis of normal adjacent to tumor transcriptomes.” Nat Commun 8(1):1077. 10.1038/s41467-017-01027-z 29057876 10.1038/s41467-017-01027-z PMC5651823

[55] Gyorffy B, Surowiak P, Budczies J, Lanczky A (2013) “ Online survival analysis software to assess the prognostic value of biomarkers using transcriptomic data in non-small-cell lung cancer.” PLoS One 8(12):e82241. 10.1371/journal.pone.0082241 24367507 10.1371/journal.pone.0082241 PMC3867325

[56] Hong OY, Kang SY, Noh EM, Yu HN, Jang HY, Kim SH et al (2022) “ Aurora kinase A induces migration and invasion by inducing epithelial-to-mesenchymal transition in colon cancer cells.” BMB Rep 55(2):87–91. 10.5483/BMBRep.2022.55.2.169 34903321 10.5483/BMBRep.2022.55.2.169 PMC8891622

[57] Fernando M, Duijf PHG, Proctor M, Stevenson AJ, Ehmann A, Vora S et al (2021) Dysregulated G2 phase checkpoint recovery pathway reduces DNA repair efficiency and increases chromosomal instability in a wide range of tumours. Oncogenesis 10(5):41. 10.1038/s41389-021-00329-8 33993200 10.1038/s41389-021-00329-8 PMC8124070

[58] Do TV, Xiao F, Bickel LE, Klein-Szanto AJ, Pathak HB, Hua X et al (2014) Aurora kinase A mediates epithelial ovarian cancer cell migration and adhesion. Oncogene 33(5):539–49. 10.1038/onc.2012.632 23334327 10.1038/onc.2012.632 PMC3640671

[59] Grisetti L, Garcia CJC, Saponaro AA, Tiribelli C, Pascut D (2024) The role of Aurora kinase A in hepatocellular carcinoma: unveiling the intriguing functions of a key but still underexplored factor in liver cancer. Cell Prolif 57(8):e13641. 10.1111/cpr.13641 38590119 10.1111/cpr.13641 PMC11294426

[60] Yin Y, Kong D, He K, Xia Q (2022) Aurora kinase A regulates liver regeneration through macrophages polarization and Wnt/beta-catenin signalling. Liver Int 42(2):468–78. 10.1111/liv.15094 34719108 10.1111/liv.15094

[61] Garcia CJC, Grisetti L, Tiribelli C, Pascut D (2024) The ncRNA-AURKA interaction in hepatocellular carcinoma: insights into oncogenic pathways, therapeutic opportunities, and future challenges. Life (Basel). 10.3390/life14111430 39598228 10.3390/life14111430 PMC11595987

[62] Reiter R, Gais P, Jutting U, Steuer-Vogt MK, Pickhard A, Bink K et al (2006) Aurora kinase A messenger RNA overexpression is correlated with tumor progression and shortened survival in head and neck squamous cell carcinoma. Clin Cancer Res 12(17):5136 – 41. 10.1158/1078-0432.CCR-05-1650 16951231

[63] Goos JA, Coupe VM, Diosdado B, Delis-Van Diemen PM, Karga C, Belien JA et al (2013) Aurora kinase A (AURKA) expression in colorectal cancer liver metastasis is associated with poor prognosis. Br J Cancer 109(9):2445 – 52. 10.1038/bjc.2013.608 PMC3817339 24104968

[64] Guo M, Lu S, Huang H, Wang Y, Yang MQ, Yang Y et al (2018) Increased AURKA promotes cell proliferation and predicts poor prognosis in bladder cancer. BMC Syst Biol 12(Suppl 7):118. 10.1186/s12918-018-0634-2 PMC6293497 30547784

[65] Zhang W, Xia D, Li Z, Zhou T, Chen T, Wu Z et al (2019) Aurora-A/ERK1/2/mTOR axis promotes tumor progression in triple-negative breast cancer and dual-targeting Aurora-A/mTOR shows synthetic lethality. Cell Death Dis 10(8):606. 10.1038/s41419-019-1855-z 31406104 10.1038/s41419-019-1855-z PMC6690898

[66] Zhang K, Chen J, Chen D, Huang J, Feng B, Han S et al (2014) “ Aurora-A promotes chemoresistance in hepatocelluar carcinoma by targeting NF-kappaB/microRNA-21/PTEN signaling pathway.” Oncotarget 5(24):12916–35. 10.18632/oncotarget.2682 25428915 10.18632/oncotarget.2682 PMC4350360

[67] Zhang H, Chen X, Jin Y, Liu B, Zhou L (2012) “ Overexpression of Aurora-A promotes laryngeal cancer progression by enhancing invasive ability and chromosomal instability.” Eur Arch Otorhinolaryngol 269(2):607–14. 10.1007/s00405-011-1629-4 21584819 10.1007/s00405-011-1629-4 PMC3259349

[68] Liu R, Jiang Z, Kong W, Zheng S, Dai T, Wang G (2021) “ A novel nine-gene signature associated with immune infiltration for predicting prognosis in hepatocellular carcinoma.” Front Genet 12:730732. 10.3389/fgene.2021.730732 34917126 10.3389/fgene.2021.730732 PMC8669621

[69] Yang T, Chen Y, Xu J, Li J, Liu H, Liu N (2022) “ Bioinformatics screening the novel and promising targets of curcumin in hepatocellular carcinoma chemotherapy and prognosis.” BMC Complement Med Ther 22(1):21. 10.1186/s12906-021-03487-9 35078445 10.1186/s12906-021-03487-9 PMC8788085

[70] Meng Z, Wu J, Liu X, Zhou W, Ni M, Liu S et al (2020) “ Identification of potential hub genes associated with the pathogenesis and prognosis of hepatocellular carcinoma via integrated bioinformatics analysis.” J Int Med Res 48(7):300060520910019. 10.1177/0300060520910019 32722976 10.1177/0300060520910019 PMC7391448

[71] Yang Z, Wu X, Li J, Zheng Q, Niu J, Li S (2021) “ CCNB2, CDC20, AURKA, TOP2A, MELK, NCAPG, KIF20A, UBE2C, PRC1, and ASPM may be potential therapeutic targets for hepatocellular carcinoma using integrated bioinformatic analysis.” Int J Gen Med 14:10185–94. 10.2147/IJGM.S341379 34992437 10.2147/IJGM.S341379 PMC8710976

[72] Jeng YM, Peng SY, Lin CY, Hsu HC (2004) “Overexpression and amplification of Aurora-A in hepatocellular carcinoma.” Clin Cancer Res 10(6):2065–71. 10.1158/1078-0432.ccr-1057-03 15041727

[73] Chen C, Song G, Xiang J, Zhang H, Zhao S, Zhan Y (2017) “ AURKA promotes cancer metastasis by regulating epithelial-mesenchymal transition and cancer stem cell properties in hepatocellular carcinoma.” Biochem Biophys Res Commun 486(2):514–20. 10.1016/j.bbrc.2017.03.075 28322787 10.1016/j.bbrc.2017.03.075

[74] Vishnoi N, Yao J (2017) “ Single-cell, single-mRNA analysis of Ccnb1 promoter regulation.” Sci Rep 7(1):2065. 10.1038/s41598-017-02240-y 28522800 10.1038/s41598-017-02240-y PMC5437063

[75] Rong MH, Li JD, Zhong LY, Huang YZ, Chen J, Xie LY et al (2022) Ccnb1 promotes the development of hepatocellular carcinoma by mediating DNA replication in the cell cycle. Exp Biol Med (Maywood) 247(5):395–408. 10.1177/15353702211049149 34743578 10.1177/15353702211049149 PMC8919315

[76] Yoshida T, Tanaka S, Mogi A, Shitara Y, Kuwano H (2004) The clinical significance of Cyclin B1 and Wee1 expression in non-small-cell lung cancer. Ann Oncol 15(2):252–6. 10.1093/annonc/mdh073 14760118 10.1093/annonc/mdh073

[77] Nimeus-Malmstrom E, Koliadi A, Ahlin C, Holmqvist M, Holmberg L, Amini RM et al (2010) Cyclin B1 is a prognostic proliferation marker with a high reproducibility in a population-based lymph node negative breast cancer cohort. Int J Cancer 127(4):961–7. 10.1002/ijc.25091 19957331 10.1002/ijc.25091

[78] Kedinger V, Meulle A, Zounib O, Bonnet ME, Gossart JB, Benoit E et al (2013) Sticky siRNAs targeting survivin and cyclin B1 exert an antitumoral effect on melanoma subcutaneous xenografts and lung metastases. BMC Cancer 13:338. 10.1186/1471-2407-13-338 23835136 10.1186/1471-2407-13-338 PMC3711931

[79] Gu J, Liu X, Li J, He Y (2019) Microrna-144 inhibits cell proliferation, migration and invasion in human hepatocellular carcinoma by targeting CCNB1. Cancer Cell Int 19:15. 10.1186/s12935-019-0729-x 30651720 10.1186/s12935-019-0729-x PMC6332595

[80] Zhuang L, Yang Z, Meng Z (2018) Upregulation of BUB1B, CCNB1, CDC7, CDC20, and MCM3 in tumor tissues predicted worse overall survival and disease-free survival in hepatocellular carcinoma patients. Biomed Res Int 2018:7897346. 10.1155/2018/7897346 30363964 10.1155/2018/7897346 PMC6186344

[81] Miki H, Setou M, Hirokawa N, Group RG, Members GSL (2003) Kinesin superfamily proteins (KIFs) in the mouse transcriptome. Genome Res 13(6B):1455–65. 10.1101/gr.984503 12819144 10.1101/gr.984503 PMC403687

[82] Hirokawa N, Tanaka Y (2015) Kinesin superfamily proteins (KIFs): various functions and their relevance for important phenomena in life and diseases. Exp Cell Res 334(1):16–25. 10.1016/j.yexcr.2015.02.016 25724902 10.1016/j.yexcr.2015.02.016

[83] Wakana Y, Villeneuve J, van Galen J, Cruz-Garcia D, Tagaya M, Malhotra V (2013) Kinesin-5/Eg5 is important for transport of CARTS from the trans-Golgi network to the cell surface. J Cell Biol 202(2):241–50. 10.1083/jcb.201303163 23857769 10.1083/jcb.201303163 PMC3718972

[84] Rapley J, Nicolas M, Groen A, Regue L, Bertran MT, Caelles C et al (2008) The NIMA-family kinase Nek6 phosphorylates the kinesin Eg5 at a novel site necessary for mitotic spindle formation. J Cell Sci 121(23):3912–21. 10.1242/jcs.035360 19001501 10.1242/jcs.035360 PMC4066659

[85] Liu X, Gong H, Huang K (2013) Oncogenic role of kinesin proteins and targeting kinesin therapy. Cancer Sci 104(6):651–6. 10.1111/cas.12138 23438337 10.1111/cas.12138 PMC7657121

[86] Hu ZD, Jiang Y, Sun HM, Wang JW, Zhai LL, Yin ZQ et al (2021) KIF11 promotes proliferation of hepatocellular carcinoma among patients with liver cancers. BioMed Res Int 2021:2676745. 10.1155/2021/2676745 33490265 10.1155/2021/2676745 PMC7801104

[87] Rath O, Kozielski F (2012) Kinesins and cancer. Nat Rev Cancer 12(8):527–39. 10.1038/nrc3310 22825217 10.1038/nrc3310

[88] Gao W, Lu J, Yang Z, Li E, Cao Y, Xie L (2024) Mitotic functions and characters of KIF11 in cancers. Biomolecules. 10.3390/biom14040386 38672404 10.3390/biom14040386 PMC11047945

[89] Garcia-Saez I, Skoufias DA (2021) Eg5 targeting agents: from new anti-mitotic based inhibitor discovery to cancer therapy and resistance. Biochem Pharmacol 184:114364. 10.1016/j.bcp.2020.114364 33310050 10.1016/j.bcp.2020.114364

[90] Yan GR, Zou FY, Dang BL, Zhang Y, Yu G, Liu X et al (2012) Genistein-induced mitotic arrest of gastric cancer cells by downregulating KIF20A, a proteomics study. Proteomics 12(14):2391–9. 10.1002/pmic.201100652 22887948 10.1002/pmic.201100652

[91] Saijo T, Ishii G, Ochiai A, Yoh K, Goto K, Nagai K et al (2006) Eg5 expression is closely correlated with the response of advanced non-small cell lung cancer to antimitotic agents combined with platinum chemotherapy. Lung Cancer 54(2):217–25. 10.1016/j.lungcan.2006.06.018 16934364 10.1016/j.lungcan.2006.06.018

[92] Wissing MD, De Morree ES, Dezentje VO, Buijs JT, De Krijger RR, Smit VT et al (2014) Nuclear Eg5 (kinesin spindle protein) expression predicts docetaxel response and prostate cancer aggressiveness. Oncotarget 5(17):7357–67. 10.18632/oncotarget.1985 25277178 10.18632/oncotarget.1985 PMC4202128

[93] Daigo K, Takano A, Thang PM, Yoshitake Y, Shinohara M, Tohnai I et al (2018) Characterization of KIF11 as a novel prognostic biomarker and therapeutic target for oral cancer. Int J Oncol 52(1):155 – 65. 10.3892/ijo.2017.4181 PMC5743338 29115586

[94] Zhang J, Wei Z, Qi X, Jiang Y, Liu D, Liu K (2023) Kinesin family member 11 promotes progression of hepatocellular carcinoma via the OCT4 pathway. Funct Integr Genomics 23(3):284. 10.1007/s10142-023-01209-7 37648881 10.1007/s10142-023-01209-7

[95] Medina PP, Slack FJ (2008) Micrornas and cancer: an overview. Cell Cycle 7(16):2485–92. 10.4161/cc.7.16.6453 18719380 10.4161/cc.7.16.6453

[96] Lewis BP, Burge CB, Bartel DP (2005) Conserved seed pairing, often flanked by adenosines, indicates that thousands of human genes are microrna targets. Cell 120(1):15–20. 10.1016/j.cell.2004.12.035 15652477 10.1016/j.cell.2004.12.035

[97] Tavazoie SF, Alarcon C, Oskarsson T, Padua D, Wang Q, Bos PD et al (2008) Endogenous human micrornas that suppress breast cancer metastasis. Nature 451(7175):147–52. 10.1038/nature06487 18185580 10.1038/nature06487 PMC2782491

[98] Benetti R, Gonzalo S, Jaco I, Munoz P, Gonzalez S, Schoeftner S et al (2008) A mammalian microRNA cluster controls DNA methylation and telomere recombination via Rbl2-dependent regulation of DNA methyltransferases. Nat Struct Mol Biol 15(9):998. 10.1038/nsmb0908-998b 18769471

[99] Sinkkonen L, Hugenschmidt T, Berninger P, Gaidatzis D, Mohn F, Artus-Revel CG et al (2008) Micrornas control de novo DNA methylation through regulation of transcriptional repressors in mouse embryonic stem cells. Nat Struct Mol Biol 15(3):259–67. 10.1038/nsmb.1391 18311153 10.1038/nsmb.1391

[100] Qiu P, Guo Q, Yao Q, Chen J, Lin J (2021) Hsa-mir-3163 and CCNB1 may be potential biomarkers and therapeutic targets for androgen receptor positive triple-negative breast cancer. PLoS One 16(11):e0254283. 10.1371/journal.pone.0254283 34797837 10.1371/journal.pone.0254283 PMC8604295

[101] Yang B, Wang C, Xie H, Wang Y, Huang J, Rong Y et al (2019) Microrna-3163 targets ADAM-17 and enhances the sensitivity of hepatocellular carcinoma cells to molecular targeted agents. Cell Death Dis 10(10):784. 10.1038/s41419-019-2023-1 31611551 10.1038/s41419-019-2023-1 PMC6791891

[102] Rojas F, Hernandez ME, Silva M, Li L, Subramanian S, Wilson MJ et al (2015) The oncogenic response to MiR-335 is associated with cell surface expression of membrane-type 1 matrix metalloproteinase (MT1-MMP) activity. PLoS One 10(7):e0132026. 10.1371/journal.pone.0132026 26204513 10.1371/journal.pone.0132026 PMC4512721

[103] Zhang JK, Li YS, Zhang CD, Dai DQ (2017) Up-regulation of CRKL by microrna-335 methylation is associated with poor prognosis in gastric cancer. Cancer Cell Int 17:28. 10.1186/s12935-017-0387-9 28239297 10.1186/s12935-017-0387-9 PMC5314703

[104] Dohi O, Yasui K, Gen Y, Takada H, Endo M, Tsuji K et al (2013) Epigenetic silencing of miR-335 and its host gene MEST in hepatocellular carcinoma. Int J Oncol 42(2):411–8. 10.3892/ijo.2012.1724 23229728 10.3892/ijo.2012.1724 PMC3583616

[105] Lopez-Serra P, Esteller M (2012) DNA methylation-associated silencing of tumor-suppressor microRNAs in cancer. Oncogene 31(13):1609–22. 10.1038/onc.2011.354 21860412 10.1038/onc.2011.354 PMC3325426

