# Supplementary material for: Network-based discovery of gene-miRNA interactions associated with hepatocellular carcinoma
Source: Ir J Med Sci. 2026 Apr 2;195(3):1253–65. doi: 10.1007/s11845-026-04303-4 (PMC13342284; doi:10.1007/s11845-026-04303-4)
Supplement: Supplementary file 1 — Supplementary Material 1 (DOCX 36.1 KB) [file 11845_2026_4303_MOESM1_ESM.docx]

**Table S1.** List of differentially expressed genes (DEGs) distributions from each dataset. **GEO**; gene expression omnibus.

| GEO | Total of DEGs | Upregulated DEGs | Downregulated DEGs |
| --- | --- | --- | --- |
| GSE263134 | 1679 | 853 | 826 |
| GSE242797 | 2768 | 923 | 1845 |
| GSE202069 | 2604 | 1625 | 979 |
| GSE169287 | 2359 | 1274 | 1085 |
| GSE169289 | 3131 | 1685 | 1446 |
| GSE135631 | 3118 | 1730 | 1388 |
| GSE216613 | 3787 | 1985 | 1802 |
| GSE214846 | 2567 | 1349 | 1218 |
| GSE113617 | 4117 | 2626 | 1491 |
| GSE185799 | 4471 | 2325 | 2146 |
| GSE159220 | 3583 | 2151 | 1432 |

**Table S2.** Common up-regulated and down-regulated DEGs in GEOs derived from HCC tumor tissues and adjacent non-tumor tissues. **HCC**; hepatocellular carcinoma, **DEGs**; differentially expressed genes, **GEO**; gene expression omnibus.

| Shared up-regulated (*p*-value <0.05, \|log2FC\| ≥ 1) genes in all GEOs | | | | |
| --- | --- | --- | --- | --- |
| *ANLN* | *ARHGAP11A* | *ARHGAP11B* | *ASF1B* | *ASPM* |
| *AURKA* | *AURKB* | *BCL9* | *BUB1* | *BUB1B* |
| *CABYR* | *CCNA2* | *CCNB1* | *CCNB2* | *CCNF* |
| *CD34* | *CDC20* | *CDC25A* | *CDC25C* | *CDC45* |
| *CDC6* | *CDCA5* | *CDCA8* | *CDK1* | *CDKN2A* |
| *CENPE* | *CENPF* | *CENPİ* | *CENPL* | *CENPU* |
| *CHEK1* | *CKAP2L* | *CLSPN* | *DEPDC1B* | *DNAJC6* |
| *DTL* | *E2F1* | *ECT2* | *EXO1* | *EZH2* |
| *FAM83D* | *FANCD2* | *FANCİ* | *FBXL18* | *FLVCR1* |
| *FOXM1* | *GİNS1* | *GMNN* | *GNAZ* | *GPC3* |
| *GTSE1* | *HELLS* | *HROB* | *İGF2BP3* | *İQGAP3* |
| *KİF11* | *KİF14* | *KİF15* | *KİF18B* | *KİF20A* |
| *KİF20B* | *KİF2C* | *KİF4A* | *KİFC1* | *KNTC1* |
| *KPNA2* | *LAPTM4B* | *LİN9* | *LMNB1* | *MCM10* |
| *MCM2* | *MCM3* | *MCM4* | *MCM8* | *MDK* |
| *MELK* | *MKİ67* | *MSTO1* | *MYBL2* | *NCAPG* |
| *NCAPG2* | *NCAPH* | *NDC80* | *NOX4* | *NUSAP1* |
| *ORC1* | *ORC6* | *PAFAH1B3* | *PARPBP* | *PLCB1* |
| *PLK1* | *PLVAP* | *POLQ* | *PRC1* | *PRR11* |
| *PSPH* | *PTP4A3* | *PTTG1* | *RAB3B* | *RACGAP1* |
| *RAD54L* | *RECQL4* | *RNASEH2A* | *ROBO1* | *RRM2* |
| *SKA1* | *SLC26A6* | *SMC4* | *SPAG5* | *SPC24* |
| *SPC25* | *SQLE* | *STİL* | *STMN1* | *TİCRR* |
| *TMEM106C* | *TONSL* | *TOP2A* | *TRAİP* | *TROAP* |
| *TUBG1* | *UBE2C* | *UBE2S* | *UCK2* | *WDR62* |
| *WDR76* |  |  |  |  |
| Shared down-regulated (*p*-value <0.05, \|log2FC\| ≤ 1) genes in all GEOs | | | | |
| *A2M* | *AADAT* | *ACAA2* | *ACAD11* | *ACSM3* |
| *ADRA1A* | *ALDH2* | *ALDH8A1* | *ASS1* | *BCKDHB* |
| *BMPER* | *C1R* | *CDHR2* | *CXCL12* | *CYP26A1* |
| *DCN* | *DHODH* | *DLG2* | *DMGDH* | *DPF3* |
| *EPHX2* | *ETFDH* | *GHR* | *GNAO1* | *GOT2* |
| *GPM6A* | *HMGCL* | *LİFR* | *MAN1C1* | *MASP1* |
| *MAT1A* | *MT2A* | *N4BP2L1* | *OİT3* | *PHYHD1* |
| *PİTPNM3* | *PLAC8* | *RASGEF1B* | *SLC25A47* | *SLC28A1* |
| *SORL1* | *STEAP3* | *TİMD4* | *TRPV3* | *TUBE1* |
| *VİPR1* | *ZFP1* |  |  |  |

**Table S3.** Top 20 genes evaluated in the PPI network using five calculation methods and employing CytoHubba in Cytoscape.

The overlap hub genes in the top 20 by five ranked methods respectively in cytoHubba are highlighted in bold. **MCC**: Maximal Clique Centrality, **MNC**: Maximum Neighborhood Component, **EPC**: Edge Percolated Component.

| **MCC** | **MNC** | **Degree** | **Closeness** | **EPC** |
| --- | --- | --- | --- | --- |
| FANCD2 | CDK1 | CDK1 | CDK1 | CDK1 |
| ANLN | **CCNB1** | **CCNB1** | CCNA2 | CDCA8 |
| CDC6 | CCNA2 | CCNA2 | **CCNB1** | CCNA2 |
| **AURKA** | TOP2A | TOP2A | TOP2A | **KIF11** |
| MYBL2 | BUB1B | BUB1B | **AURKA** | BUB1 |
| GMNN | **KIF11** | **KIF11** | BUB1B | **CCNB1** |
| NCAPH | CDC45 | CDC45 | CDC45 | CCNB2 |
| NCAPG | CDC20 | CDC20 | PLK1 | TOP2A |
| **CCNB1** | PLK1 | PLK1 | CDC20 | CDC45 |
| **KIF11** | **AURKA** | **AURKA** | AURKB | KIF20A |
| LMNB1 | CDCA8 | CDCA8 | RRM2 | PLK1 |
| NDC80 | AURKB | AURKB | **KIF11** | EXO1 |
| MCM4 | RRM2 | RRM2 | CDCA8 | AURKB |
| GINS1 | ASPM | ASPM | BUB1 | CDCA5 |
| DEPDC1B | BUB1 | BUB1 | ASPM | KIF2C |
| MCM2 | CCNB2 | CENPF | CENPF | CENPF |
| CENPE | EXO1 | CCNB2 | EXO1 | RRM2 |
| CLSPN | KIF2C | EXO1 | MCM10 | CDC20 |
| CENPU | CENPF | KIF2C | KIF2C | **AURKA** |
| TICRR | MCM10 | MCM10 | CCNB2 | FOXM1 |

**Table S4.** GO (Gene Ontology) and KEGG (Kyoto Encyclopedia of Genes and Genomes) pathway enrichment analysis results of genes found to be differentially expressed (*p*-value <0.05, |log2FC| ≥ 1 and |log2FC| ≤ 1) between HCC tumor tissues compared with adjacent non-tumor tissues. The top 10 significant GO and pathway terms were listed. **HCC**; hepatocellular carcinoma, **MF**; Molecular Function, **BP**; Biological Process, and **CC**; Cellular Component.

|  | Term ID | Term description | *p-*value | *q*-value FDR B&H | Hit in Query List |
| --- | --- | --- | --- | --- | --- |
| MF: Molecular Function | | | | | |
|  | GO:0019901 | Protein kinase binding | 8,33E-05 | 2,39E-03 | *AURKA, KIF11, CCNB1* |
|  | GO:0019900 | kinase binding | 1,14E-04 | 2,39E-03 | *AURKA, KIF11, CCNB1* |
|  | GO:0035175 | histone H3S10 kinase activity | 6,01E-04 | 8,41E-03 | *AURKA* |
|  | GO:0044389 | ubiquitin-like protein ligase binding | 9,50E-04 | 9,98E-03 | *AURKA, CCNB1* |
|  | GO:0005113 | patched binding | 1,20E-03 | 1,01E-02 | *CCNB1* |
|  | GO:0140996 | histone H3 kinase activity | 1,80E-03 | 1,26E-02 | *AURKA* |
|  | GO:0061575 | cyclin-dependent protein serine/threonine kinase activator activity | 2,40E-03 | 1,34E-02 | *CCNB1* |
|  | GO:0008574 | plus-end-directed microtubule motor activity | 2,55E-03 | 1,34E-02 | *KIF11* |
|  | GO:0035173 | histone kinase activity | 4,20E-03 | 1,96E-02 | *AURKA* |
|  | GO:0019900 | kinase binding | 1,14E-04 | 2,39E-03 | *AURKA, KIF11, CCNB1* |
| BP: Biological Process | | | | | |
|  | GO:0007052 | mitotic spindle organization | 3,85E-07 | 1,03E-04 | *AURKA, KIF11, CCNB1* |
|  | GO:1902850 | microtubule cytoskeleton organization involved in mitosis | 7,33E-07 | 1,03E-04 | *AURKA, KIF11, CCNB1* |
|  | GO:0007051 | spindle organization | 1,26E-06 | 1,03E-04 | *AURKA, KIF11, CCNB1* |
|  | GO:0007100 | mitotic centrosome separation | 1,28E-06 | 1,03E-04 | *AURKA, KIF11* |
|  | GO:0051299 | centrosome separation | 1,69E-06 | 1,08E-04 | *AURKA, KIF11* |
|  | GO:0140014 | mitotic nuclear division | 3,56E-06 | 1,90E-04 | *AURKA, KIF11, CCNB1* |
|  | GO:0090231 | regulation of spindle checkpoint | 4,58E-06 | 2,04E-04 | *AURKA, CCNB1* |
|  | GO:0098813 | nuclear chromosome segregation | 5,10E-06 | 2,04E-04 | *AURKA, KIF11, CCNB1* |
|  | GO:0007059 | chromosome segregation | 1,14E-05 | 4,05E-04 | *AURKA, KIF11, CCNB1* |
|  | GO:0000280 | nuclear division | 1,52E-05 | 4,43E-04 | *AURKA, KIF11, CCNB1* |
| CC: Cellular Component | | | | | |
|  | GO:0000922 | spindle pole | 9,30E-07 | 5,58E-05 | *AURKA, KIF11, CCNB1* |
|  | GO:0005819 | spindle | 1,14E-05 | 3,41E-04 | *AURKA, KIF11, CCNB1* |
|  | GO:0005876 | spindle microtubule | 5,01E-05 | 1,00E-03 | *AURKA, KIF11* |
|  | GO:0005875 | microtubule associated complex | 1,76E-04 | 2,15E-03 | *AURKA, KIF11* |
|  | GO:0000776 | kinetochore | 2,22E-04 | 2,15E-03 | *AURKA, CCNB1* |
|  | GO:0000779 | condensed chromosome, centromeric region | 2,53E-04 | 2,15E-03 | *AURKA, CCNB1* |
|  | GO:0072686 | mitotic spindle | 2,74E-04 | 2,15E-03 | *AURKA, KIF11* |
|  | GO:0097125 | cyclin B1-CDK1 complex | 2,87E-04 | 2,15E-03 | *CCNB1* |
|  | GO:0015630 | microtubule cytoskeleton | 3,72E-04 | 2,48E-03 | *AURKA, KIF11, CCNB1* |
|  | GO:0099080 | supramolecular complex | 4,21E-04 | 2,53E-03 | *AURKA, KIF11, CCNB1* |

| KEGG Pathway | | | | | | |
| --- | --- | --- | --- | --- | --- | --- |
|  | M16817 | oocyte meıosıs | 1,90E-04 | 2,28E-03 | *AURKA, CCNB1* |  |
|  | M49022 | Hıv vpr to wee1 cell cycle g2m | 1,06E-03 | 4,37E-03 | *CCNB1* |  |
|  | M47574 | Wee1 cell cycle g2 m | 1,49E-03 | 4,37E-03 | *CCNB1* |  |
|  | M47586 | atr p21 cell cycle g2 m | 1,91E-03 | 4,37E-03 | *CCNB1* |  |
|  | M47572 | cdc25 cell cycle g2 m | 2,13E-03 | 4,37E-03 | *CCNB1* |  |
|  | M47573 | hıv vpr to cdc25 cell cycle g2m | 2,34E-03 | 4,37E-03 | *CCNB1* |  |
|  | M47887 | cenpe ınteractıon wıth ndc80 complex | 2,55E-03 | 4,37E-03 | *AURKA* |  |
|  | M47910 | mıcrotubule depolymerızatıon | 4,89E-03 | 6,52E-03 | *AURKA* |  |
|  | M47900 | kınetochore fıber organızatıon | 4,89E-03 | 6,52E-03 | *AURKA* |  |
|  | M6370 | p53 sıgnalıng pathway | 1,44E-02 | 1,73E-02 | *CCNB1* |  |
